# In the search for the perfect prompt in medical AI queries

**DOI:** 10.3389/frai.2025.1689178

**Published:** 2025-11-17

**Authors:** Florian Berghea, Elena Camelia Berghea, Cristina Octaviana Daia, Diana Ciuc, Gabi Valeriu Dinca

**Affiliations:** 1 Sfanta Maria Clinical Hospital, Carol Davila University of Medicine and Pharmacy, Bucharest, Romania; 2 Marie S. Curie Emergency Children’s Clinical Hospital, Carol Davila University of Medicine and Pharmacy, Bucharest, Romania; 3 “CF 2" Clinical Hospital, Bucharest, Romania

**Keywords:** artificial intelligence, prompt engineering, medical AI, generated responses, performance evaluation, AI ethics, general public, medical information

## Abstract

The evaluation of medical Artificial Intelligence (AI) systems presents significant challenges, with performance often varying drastically across studies. This narrative review identifies prompt quality—the way questions are formulated for the AI—as a critical yet under-recognized variable influencing these outcomes. The analysis explores scientific literature published between January 2018 and August 2025 to investigate the impact of prompt engineering on the perceived accuracy and reliability of conversational AI in medicine. Results reveal a “performance paradox,” where AI sometimes surpasses human experts in controlled settings yet underperforms in broader meta-analyses. This inconsistency is strongly linked to the type of prompt used. Critical concerns are highlighted, such as “prompting bias,” which may invalidate study conclusions, and AI “hallucinations” that generate dangerously incorrect information. Furthermore, a significant gap exists between the optimal prompts formulated by experts and the natural queries of the general public, raising issues of safety and health equity. In the end we were interested in finding out what the optimal balance existed between the complexity of a prompt and the value of the generated response, and, in this context, whether we could attempt to define a path toward identifying the best possible prompt.

## 1 Introduction

The emergence of what is generically called “artificial intelligence” has created a cultural shock throughout society due to the unprecedent abilities of AI tools to communicate in an almost natural way with users. This mode of communication has led both specialists and the general public to believe that the simple act of obtaining a response from AI to a formulated question (in more sophisticated terms, the question is called a prompt) means that both the question and, especially, its meaning and context have been fully deciphered by the AI. A wide range of studies were subsequently published evaluating the various dimensions of AI systems, studies in which technicians with expertise in AI systems engineering were only slightly involved. Although these studies concluded with the most attractive findings, we retrospectively note that these conclusions might be called into question as long as the research teams did not consider the importance of how communication with the AI system was carried out and, especially, how the prompts used were formulated.

When approaching the specialized literature in an attempt to understand if and to what extent we can rely on medical AI systems, we identify often contradictory results, with studies in which the same AI system generates valuable responses that surpass physicians and others where the results are much inferior. The extremely rapid evolution of these systems creates the premises to accept this variability, attributing it to the leaps (sometimes major) from one moment to the next, but we have no way to truly measure this explanation. Another variable that is more often considered is the approach to the AI system: in strictly controlled contexts (a kind of equivalent of “*in vitro*” studies) or in the absence of any constraints (let us say these could be the equivalent of “*in vivo*” studies).

There are several definitions of prompts, but it is generally accepted that a prompt is a natural language text that describes the task an AI model should perform (Liu et al., 2021). In this context, prompt engineering is defined as the practice of designing, formulating, and adjusting instructions (prompts) in natural language or other formats, with the aim of guiding large language models (LLMs) in generating relevant, accurate, and useful responses for the desired task (Liu et al., 2021; Reynolds and K, 2021). Due to its complexity and importance, prompt engineering has become an emerging discipline in itself with first-rank implications. This refers not only to the correct formulation of questions but also to how AI systems process and understand information to generate adequate responses. In medicine, its applications range from decision support for doctors and administrative assistance to improving communication with patients, research, medical education, and public health.

Discussions about prompting techniques have appeared relatively recently in the world of those who use AI systems without having an adequate software engineering background. Different results generated from one prompt to another have raised the question of the extent to which the way we communicated with the AI systems was the most appropriate, questioning the validity of the results. It is problematic that in already published medical studies the reporting of baseline non-prompt response levels is mediocre—Zaghir et al. identified 61% of studies lacking such reporting (Zaghir et al., 2024). This hinders efforts to optimize the use of AI systems and their correct and appropriate application. The quality of prompts directly influences patient safety and the standard of medical care through the response generated by the AI system. A poorly formulated medical prompt can lead to incorrect or incomplete responses, with harmful potential, while a well-designed prompt can unlock the full potential of the AI system. A major challenge is that most end-users do not possess the technical or medical knowledge necessary to formulate an optimal prompt and to critically evaluate the received response. Recent scientific literature reflects a complex picture, with studies demonstrating excellent AI effectiveness, while others highlight its inferiority compared to human experts. This performance fluctuation can be attributed to multiple and interconnected factors: the quality of prompts, the complexity of the questions, the characteristics of the target population, the cultural and linguistic context, as well as the analytical framework used. The fundamental challenge lies in the gap between optimal prompts, which require advanced knowledge, and the natural way of querying by the general public.

The specific objectives of this analysis were to understand the extent to which we can speak of a prompting bias when LLM performance on medical questions was evaluated or when AI performance was compared with that of medical experts. We tried to study how the relationship between prompt quality and the accuracy/utility of the response was reflected in the scientific literature. Finally, we aimed to formulate recommendations for future research and practical implementation focused on prompt technology.

## 2 Materials and methods

For this narrative review, we conducted a structured literature search of PubMed, Scopus, and arXiv to identify relevant studies published between January 2018 and August 2025. The final search was conducted on September 29, 2025. Search strategies combined MeSH terms and free-text keywords related to LLMs, prompt design, and medical applications. An example search string used is: (“large language models” OR “conversational AI” OR “ChatGPT”) AND (“prompt engineering” OR “prompt design”) AND (“medical” OR “healthcare” OR “clinical accuracy”). Inclusion criteria targeted original articles, systematic reviews, or meta-analyses in English that evaluated the performance of LLMs on medical tasks with an explicit analysis of prompts. Exclusion criteria included opinion pieces, non-medical studies, or articles lacking details on querying methods. Two reviewers independently screened titles and abstracts, with consensus resolution for discrepancies. Eligible studies included experimental, observational, systematic, meta-analytic, and technical analyses, mostly from North America and Europe, with limited data from Asia and Africa.

## 3 Results and discussion

### 3.1 Prompt engineering

Prompt engineering refers to the design and refinement of instructions that guide LLMs in performing various tasks. The techniques vary in complexity, from basic prompts (zero-shot), which consist of a direct question, to structured techniques (few-shot), which provide context and examples. Other techniques include “chain-of-thought prompting,” where the AI is asked to reason step-by-step, and “role-playing prompting,” where the AI adopts a specific role (e.g., a medical specialist). A critical aspect identified in the literature is the gap between theoretical, optimized prompts and the natural ones used by the general public. An optimized prompt requires a level of medical knowledge and a logical formulation that most patients do not possess, especially in a state of anxiety. One of the most comprehensive systematizations of prompts is carried out by Patil et al. (Patil et al., 2024). In Table 1 we present the characteristics of these prompts and, for better understanding. Supplementary Appendix 1 provides examples of prompts generated based on three published scientific materials (Onose et al., 2017; Onose et al., 2021; Berghea et al., 2021).

**Table 1:** Types of prompts (Patil et al., 2024).

Zero-shot
*Description*: A prompt that provides a task or question without any examples (zero-shot) or additional context.
*Usefulness*: Useful for quickly generating responses to simple, straightforward queries or task.
Few-shot
*Description*: A prompt that includes a few examples (few-shot) or demonstrations of the desired output before presenting the actual task or question.
*Usefulness*: Helps the AI model better understand the expected format, style, and content of the response.
Ask me anything
*Description*: An open-ended prompt that encourages the AI model to respond to a wide range of questions or tasks related to a specific domain or topic.
*Usefulness*: Enables clinicians to quickly access information and insights on various aspects of patient care, from diagnosis to treatment and beyond.
Least-to-most
*Description*: A prompt that breaks down a complex task into smaller, incremental steps, gradually guiding the AI model toward the final desired output.
*Usefulness*: Useful for tackling more challenging or multi-faceted problems in healthcare, such as developing comprehensive treatment plans.
Role assignment
*Description*: A prompt that assigns a specific role or expertise to the AI model, encouraging it to respond as if it were a particular type of entity or expert.
*Usefulness*: Helps clinicians obtain insights and recommendations from different viewpoints, such as those of specialists or patient advocates.
Tone
*Description*: A prompt that specifies the desired tone, style, or level of complexity for the AI-generated response.
*Usefulness*: Enables clinicians to tailor the output to the intended audience or purpose, such as creating patient-friendly explanations or generating professional medical reports.
Contextual priming
*Description*: A prompt that provides relevant background information or context before presenting the main task or question.
*Usefulness*: Helps the AI model generate more accurate and context-aware responses by considering factors such as patient demographics, medical history, or clinical setting.

### 3.2 Evaluation of prompt engineering techniques

Prompt complexity (PC) can be defined as a multivariate construct that integrates dimensions of informational structure and content. It depends on factors such as the total length expressed in tokens, the number of examples provided (k-shots), the degree of structural complexity (formatting, hierarchical organization), the number of explicit reasoning steps used in “Chain-of-Thought” (CoT) techniques, as well as the human cognitive effort required for design and validation. Such a conceptualization enables an objective quantification of complexity and facilitates comparisons across different prompting strategies (Brown et al., 2020). The value of the response (VR) can be defined as a composite indicator of output quality, assessed through a set of criteria specific to the medical domain. These include factual accuracy, relevance to the clinical task, logical coherence of the reasoning, adherence to the required format and style, robustness to minor input variations, and safety, understood as the absence of potentially harmful information. Naturally, each of these variables carries a different weight in determining the final value of the response; in the medical context, value cannot be reduced to a single metric but instead requires a holistic evaluation that accounts for the domain’s specificity.

To move beyond a purely conceptual evaluation, we propose a quantification framework for PC and VR. Prompt Complexity (PC) can be modeled as a composite score: PC=w1(Length)+w2(Examples)+w3(Structure)+w4(CognitiveEffort) where Length corresponds to the number of tokens, Examples to the k-shot instances used in few-shot prompting, Structure to the presence of formatting elements (e.g., markdown), explicit reasoning steps (chain-of-thought), or role assignments, and Cognitive Effort to the time and expertise required to design the prompt.

Value of Response (VR) can also be defined as a composite indicator, weighted according to the medical context: VR=w1(Accuracy)+w2(Relevance)+w3(Coherence)+w4(Safety) where Accuracy denotes the factual correctness of information verified against trusted medical sources, Relevance indicates how directly the output addresses the clinical task, Coherence reflects the internal logic and fluency of the reasoning, and Safety refers to the absence of potentially harmful content. To facilitate future operationalization, we suggest a preliminary scoring framework for both constructs. Each Prompt Complexity (PC) dimension could be rated on a 1–5 scale, with indicative equal weights of 0.25 each. Similarly, Value of Response (VR) dimensions may be rated 1–5, with proposed provisional medical-use weights of 0.35, 0.25, 0.20, and 0.20, respectively. These formulations provide a rigorous and comparable basis for evaluating different prompting strategies (Rajpurkar et al., 2022). Considering the complexity of prompts, it is expected that simple, natural ones would offer inferior results to complex ones. Contrary to expectations, recent studies question the benefits of complex prompting techniques. An evaluation by Jeon and Kim (2025) tested five LLMs (GPT-4o-mini, GPT-3.5-turbo, o1-mini, Gemini-1.5-Flash, Gemini-1.0-pro) using four CoT techniques (Jeon and Kim, 2025). Traditional CoT, ReAct CoT, Interactive CoT, and Self-Consistency prompts have been used in the study. Traditional CoT guides responses step by step (Wei et al., 2023; Savage et al., 2024). ReAct CoT combines reasoning and action in iterative cycles of thought, action, and observation (Yao et al., 2023). Interactive CoT tests metacognition by monitoring reasoning, tracking thought progression, and handling uncertainty. Self-Consistency CoT generates multiple reasoning paths and selects the most coherent outcome (Wang et al., 2023). The authors recorded no statistically significant differences between the prompting methods in the tested datasets concluded that the model’s architecture and the nature of the dataset have a greater influence on the results than the prompting technique. The study suggests that complex prompting methods do not significantly improve performance compared to simple ones.

In another study, Rebitschek et al. evaluated three LLMs (ChatGPT, Gemini, LesChat) using prompts of varying complexity (Rebitschek et al., 2025). Even with optimized prompts, none of the models reached a 50% threshold of compliance with standards for evidence-based medical information. However, the authors demonstrated that a simple “boost” type intervention—reminding users to ask about the potential consequences of medical actions—can improve the quality of the generated information, which indicates that not only the quality of the prompts is important in generating valuable responses.

### 3.3 A balance between prompt complexity and response value

**Figure 1:**
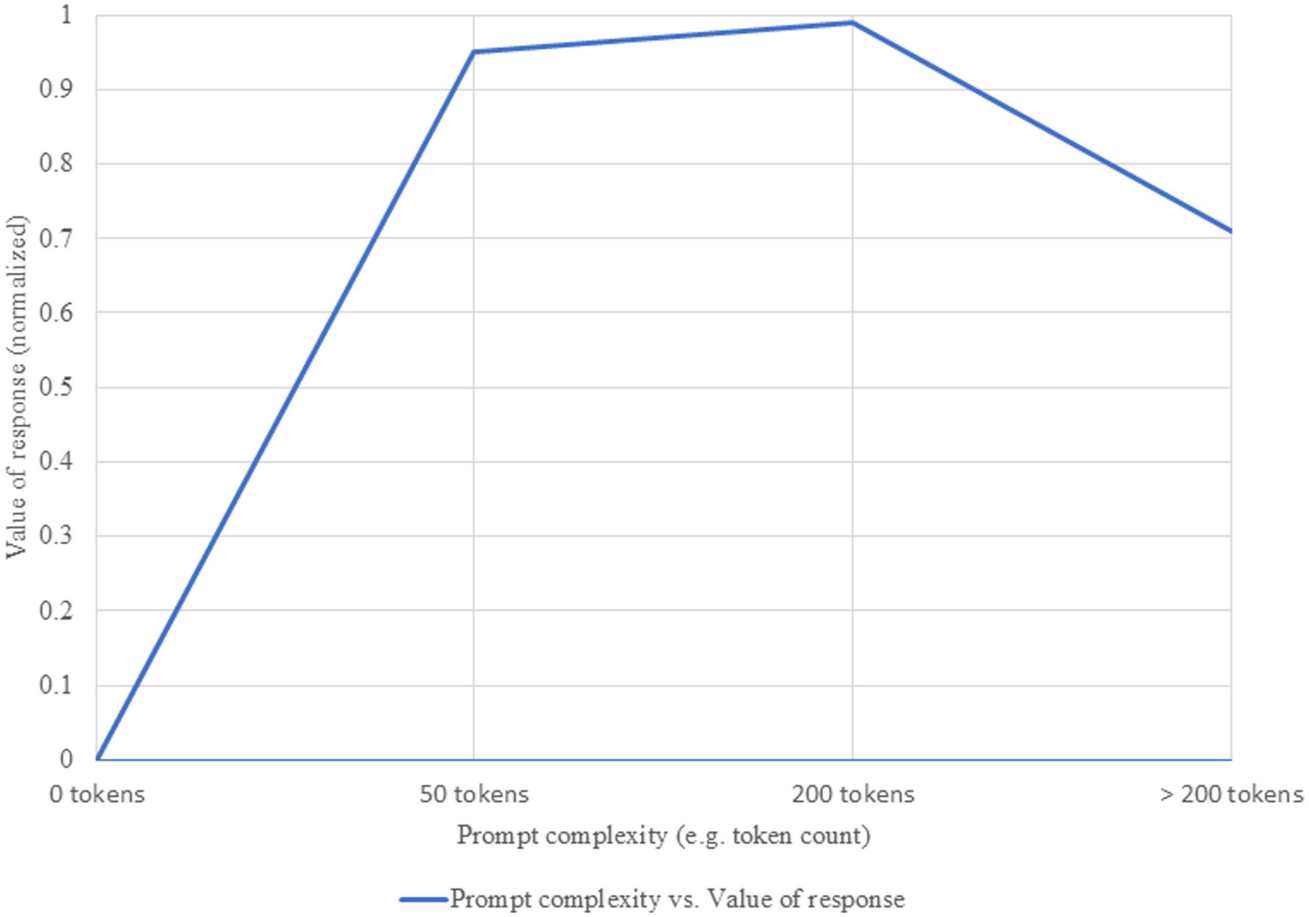
Relationship between prompt complexity and value of response. Increasing prompt complexity is associated with higher informational richness and accuracy of generated responses, up to an optimal threshold beyond which marginal gains diminish.

For many reasons (cost, time, computational resources), optimizing the effort required to obtain a valuable response from the system is a primary research objective. Our evaluation identified a non-linear relationship between prompt complexity and response value, which follows a three-phase curve: rapid growth, performance plateau, and decline with excessive complexity (Savage et al., 2024). These phases, heuristically defined based on observations from the literature, can be approximated by token ranges. In the initial rapid growth phase (approx. 0–50 tokens), each additional element brings substantial gains. In the plateau phase (approx. 50–200 tokens), an optimal level for most medical applications is reached. The third phase (>200 tokens) can lead to counterproductive results (see Figure 1). It is important to note that we cannot speak of a universal prompt value—one with qualities that would make it optimal for application across all LLMs. The inflection point defined earlier varies from model to model, favoring either more complex or, conversely, simpler prompts. Sophisticated, state-of-the-art models can benefit from more complex prompts, with an optimal point around 150–250 tokens, while smaller models reach peak performance at 50–100 tokens (Touvron et al., 2023). Another determinant is task complexity: for simple tasks, minimal prompts are sufficient, whereas for complex tasks, such as differential diagnosis, more elaborate prompts are more effective (Singhal et al., 2023). Resource constraints must also be considered—when resources are limited, the optimal point shifts toward lower levels of complexity, where efficiency is prioritized over maximum performance (OpenAI et al., 2024).

### 3.4 Comparative performance: AI versus physicians

A pressing research question in this area concerns the ability of AIs to provide answers comparable to those of medical specialists. The conclusions are divergent. A meta-analysis by Takita et al., which included 83 studies, showed that the average diagnostic accuracy of AI models is 52.1% (Takita et al., 2025). No significant performance difference was found between AI and non-expert physicians (difference of 0.6%, *p* = 0.93), but AI models were inferior to expert physicians, with an accuracy difference of 15.8% (*p* = 0.007). However, an important limitation of this meta-analysis is the absence of a detailed evaluation of the prompting methods used in the included studies. Unfortunately, most of the aggregated studies did not report prompts in a standardized manner, which prevented stratification of AI performance according to this factor. This limitation reduces the precision of the overall conclusions and suggests that future research, particularly meta-analyses, should incorporate prompt quality as an independent variable to improve the understanding of AI capabilities and limitations in medicine.

In contrast, the AMIE (Articulate Medical Intelligence Explorer) study, conducted by Tu et al., reported positive results for their conversational AI system, designed for diagnostic dialogues (Tu et al., 2025). In a randomized, double-blind study that simulated text-based medical consultations with 20 primary care physicians, AMIE demonstrated superior diagnostic accuracy and was rated better by specialist physicians and patient-actors on most performance axes, including history taking, management, communication, and empathy. These results suggest that, under controlled conditions and with optimized prompts, AI systems can surpass the performance of primary care physicians. However, the authors acknowledge the study’s limitations, such as the extremely controlled environment and the use of text chat, which are not representative of real clinical interactions.

### 3.5 Quality and safety of AI responses

The quality and safety of AI-generated responses are a major concern. Studies have revealed significant problems of consistency and accuracy, with AI occasionally providing completely different answers to the same question. A particularly serious problem is that of “AI hallucinations”—the generation of false information or fictitious references. Shiferaw et al. documented cases where ChatGPT provided fictitious references and citations to support its claims (Shiferaw et al., 2024). In one example, when asked about the reasons why SGLT2 inhibitors would be preferred over GLP-1 agonists, ChatGPT incorrectly stated that the former “can lead to a modest increase in weight” and cited an article as a source. Upon verification, the article demonstrated that patients treated with SGLT2 inhibitors lost weight. Calculation errors, incorrect units of measurement, and the use of outdated medical guidelines were also identified, errors with harmful potential. However, these effects have not been correlated with the type and quality of prompts, which leaves room for future research.

Reducing the risks associated with conversational AI in medicine requires a combination of robust technical and methodological safeguards. One of the most effective approaches is Retrieval-Augmented Generation (RAG), which grounds model outputs in external, trusted knowledge bases such as PubMed, UpToDate, or current clinical guidelines. By retrieving relevant evidence before generating a response, RAG substantially decreases the risk of hallucinations and improves alignment with medical standards. Another strategy involves the implementation of guardrails—programmatic rules and constraints that define the system’s permissible outputs. These can prevent the model from delivering unsafe recommendations, such as precise drug dosages, and redirect users toward physician consultation when necessary. Guardrails also help filter responses that may conflict with established ethical principles. For high-stakes applications, human-in-the-loop verification remains essential. In this model, AI-generated outputs are reviewed and validated by qualified experts before being communicated to end-users, ensuring both accuracy and safety. The integration of these complementary strategies is critical for transitioning AI systems from experimental contexts to clinical practice in a safe and reliable manner.

### 3.6 Discussion

The analysis of the literature reveals a “performance paradox” (see Table 2). On the one hand, studies like AMIE show that AI can outperform physicians in controlled environments. On the other hand, comprehensive meta-analyses indicate that AI systems remain inferior to expert physicians in terms of overall diagnostic accuracy. This contradiction can be explained by several factors as following. (a) Evaluation Environment-controlled experiments with standardized scenarios favor AI systems, while evaluations in realistic conditions, with the variability of clinical interactions, favor human experience. (b) Type of Medical Expertise—comparisons with family physicians or trainees tend to be more favorable for AI, while comparisons with experienced specialists highlight the deficiencies of current systems. (c) Evaluation Criteria—evaluations focused exclusively on diagnostic accuracy may indicate higher scores for AI, while analyses that include empathy, communication, and decision-making under uncertainty will favor human clinicians.

**Table 2:** Selected relevant studies illustrating the relationship between prompt types and outcomes

Study	Task	Prompt type	Outcomes	Notes
Jeon and Kim (2025)	Medical question answering and diagnostic reasoning	Chain-of-Thought	No statistically significant difference between prompting techniques across five LLMs; overall diagnostic accuracy remained within the same range regardless of prompt complexity.	Demonstrates the performance paradox: increasing prompt sophistication did not yield better outcomes, suggesting that model architecture and dataset characteristics exert greater influence than prompt design alone.
Rebitschek et al. (2025)	Evaluation of evidence-based health information	Zero-shot and Few-shot	None of the LLMs achieved >50% compliance with evidence-based information standards, even with optimized prompts.	Highlights that prompt refinement alone cannot compensate for model limitations; however, a minimal “boost” instruction (asking about consequences of actions) substantially improved factual completeness underscoring prompt context sensitivity.
Tu et al. (2025)	Simulated diagnostic dialogues (AMIE system)	Role-assigned and Contextual prompting	AI system (AMIE) surpassed general practitioners in diagnostic accuracy, empathy, and communication quality under controlled conditions.	Exemplifies the controlled-environment advantage: performance exceeds human baseline when prompts are highly optimized and interactions tightly structured yet may not translate to real-world settings.
Shiferaw et al. (2024)	Drug therapy decision support	Zero-shot natural language prompts	High incidence of hallucinated references and pharmacologic inaccuracies (e.g., inverted conclusions on drug effects).	Illustrates the risk side of the performance paradox: simple, unverified prompts can generate confident but factually false outputs, reinforcing the need for grounded prompting and retrieval augmentation.

Although the importance of prompt quality is unanimously recognized, there is a significant gap between the optimal prompts defined in research and the way the general public interacts with AI. A specialist is expected to use more complex prompts that include relevant information, whereas individuals without medical training are likely to use simpler prompts lacking a variable amount of medically relevant details. This discrepancy represents a fundamental challenge and can perpetuate inequalities, as people with a higher level of education may obtain better answers. We must understand the importance of the inflection point on the PC/VR function and how it shifts depending on the model’s capabilities or the resources allocated. Overloading a prompt can be just as counterproductive as using a cryptic, detail-poor one.

For the end-user, this is encouraging news, as the barrier to entry for effective use of the technology may be lower than previously thought. A paradigm shift is needed: instead of educating users to formulate better prompts, we must develop AI systems capable of functioning effectively with natural, unstructured prompts, including through AI-led clarification dialogues.

This analysis has several limitations. The rapid pace of evolution in the field may mean that the most recent developments are not reflected in the evaluated literature. The methodological heterogeneity of the studies makes it difficult to compare results. Most studies focus on evaluations in controlled environments, with little data on performance under real-world usage conditions. In addition, the literature is geographically unbalanced, with most studies coming from North America and Europe, which limits the extrapolation of the results.

## 4 Conclusion and recommendations

The field of medical conversational AI is in rapid evolution, marked by both progress and challenges. The variation in study results does not reflect contradictions, but rather the complexity of evaluation and context dependency. Future activities of interest might include prospective, multicenter, long-term studies in real-world settings to evaluate health outcomes; developing standardized methodologies for performance assessment, including effect size and cost–benefit analysis; establishing robust mechanisms to detect and prevent AI hallucinations through external validation and fact-checking; and designing adaptive interfaces and prompting assistants to help users refine vague queries into clinically relevant details.

Obtaining better results through AI systems is closely tied to how prompts are generated, and improving them should be a priority not only for specialists but also for those designing interfaces between AI and the general public. Consequently, studying the relationship between prompts and AI responses is part of the broader effort to understand the so-called black box AI (Castelvecchi, 2016; Rudin, 2019), which challenges both experts and non-specialists alike.

Unfortunately, we are not in a position to define the best universally applicable prompt, and this must be understood as a starting point for each new iteration in the use of AI systems. Despite the challenges, the potential of conversational AI to democratize access to medical information is immense. Success depends on a joint effort to ensure technical reliability and an adequate governance framework.

### 4.1 Practice implications

Adaptive interfaces: AI systems should not only respond but also inquire. When receiving vague prompts (e.g., “stomach pain”), they should initiate clarification dialogues—collecting key details (location, duration, associated symptoms)—thus mimicking a medical anamnesis process.

Predefined prompts and assistants: Interfaces can offer structured templates or prompting assistants guiding users to formulate clinically relevant questions, ensuring inclusion of essential medical information.

Implicit role implementation: Public-facing AI systems should default to a “cautious and empathic medical advisor” mode, prioritizing safety and encouraging professional consultation.

RAG integration: To reduce misinformation, responses should be grounded in authoritative medical databases (e.g., PubMed, UpToDate) with transparent citation of sources.

Clinical use guidance: Clinicians should verify factual accuracy through RAG-enabled tools, while training programs should emphasize low-complexity, high-value prompt examples adaptable to routine clinical tasks.

## References

[ref1] Berghea F. Berghea C. E. Zaharia D. Trandafir A. I. Nita E. C. Vlad V. M. (2021). Residual pain in the context of selecting and switching biologic therapy in inflammatory rheumatic diseases. Front. Med. 8:712645. doi: 10.3389/fmed.2021.712645 PMC8415826 34485342

[ref2] Brown T. B. Mann B. Ryder N. Subbiah M. Kaplan J. Dhariwal P. . Language models are few-shot learners. arXiv; (2020). Available online at: http://arxiv.org/abs/2005.14165

[ref3] Castelvecchi D. (2016). Can we open the black box of AI? Nat. News 538:20. doi: 10.1038/538020a 27708329

[ref4] Jeon S. Kim H. G. (2025). A comparative evaluation of chain-of-thought-based prompt engineering techniques for medical question answering. Comput. Biol. Med. 196:110614. doi: 10.1016/j.compbiomed.2025.110614 40602316

[ref5] Liu P. Yuan W. Fu J. Jiang Z. Hayashi H. Neubig G.. Pre-train, prompt, and predict: a systematic survey of prompting methods in natural language processing. arXiv; (2021). Available online at: http://arxiv.org/abs/2107.13586

[ref6] Onose G. Anghelescu A. Blendea C. D. Ciobanu V. Daia C. O. Firan F. C. . (2021). Non-invasive, non-pharmacological/bio-technological interventions towards neurorestoration upshot after ischemic stroke, in adults-systematic, synthetic, literature review. Front. Biosci. 26, 1204–1239. doi: 10.52586/5020 34856764

[ref7] Onose G. Teoibaș-Șerban D. Popescu C. Andone I. Brumă E. Mihăescu A. . (2017). New approaches regarding the use of Actovegin^®^ in subacute/postacute/subchronic traumatic brain injury patients. Rev. Farm. 65, 772–777.

[ref8] OpenAI A. J. Adler S. Agarwal S. Ahmad L. Akkaya I. . GPT-4 technical report. arXiv; (2024). Available online at: http://arxiv.org/abs/2303.08774

[ref9] Patil R. Heston T. F. Bhuse V. (2024). Prompt engineering in healthcare. Electronics 13:2961. doi: 10.3390/electronics13152961

[ref10] Rajpurkar P. Chen E. Banerjee O. Topol E. J. (2022). AI in health and medicine. Nat. Med. 28, 31–38. doi: 10.1038/s41591-021-01614-0 35058619

[ref11] Rebitschek F. G. Carella A. Kohlrausch-Pazin S. Zitzmann M. Steckelberg A. Wilhelm C. (2025). Evaluating evidence-based health information from generative AI using a cross-sectional study with laypeople seeking screening information. Npj Digit. Med. 8:343. doi: 10.1038/s41746-025-01752-6 40490558 PMC12149300

[ref12] Reynolds L. McDonell K.. Prompt programming for large language models: beyond the few-shot paradigm. arXiv; (2021). Available online at: http://arxiv.org/abs/2102.07350

[ref13] Rudin C. (2019). Stop explaining black box machine learning models for high stakes decisions and use interpretable models instead. Nat. Mach. Intell. 1, 206–215. doi: 10.1038/s42256-019-0048-x 35603010 PMC9122117

[ref14] Savage T. Nayak A. Gallo R. Rangan E. Chen J. H. (2024). Diagnostic reasoning prompts reveal the potential for large language model interpretability in medicine. Npj Digit. Med. 7:20. doi: 10.1038/s41746-024-01010-1 38267608 PMC10808088

[ref15] Shiferaw M. W. Zheng T. Winter A. Mike L. A. Chan L. N. (2024). Assessing the accuracy and quality of artificial intelligence (AI) chatbot-generated responses in making patient-specific drug-therapy and healthcare-related decisions. BMC Med. Inform. Decis. Mak. 24:404. doi: 10.1186/s12911-024-02824-5 39719573 PMC11668057

[ref16] Singhal K. Azizi S. Tu T. Mahdavi S. S. Wei J. Chung H. W., et al. Large language models encode clinical knowledge. Nature (2023);620:172–80. doi: 10.1038/s41586-023-06291-2 37438534 PMC10396962

[ref17] Takita H. Kabata D. Walston S. L. Tatekawa H. Saito K. Tsujimoto Y. (2025). A systematic review and meta-analysis of diagnostic performance comparison between generative AI and physicians. Npj Digit. Med. 8:175. doi: 10.1038/s41746-025-01543-z 40121370 PMC11929846

[ref18] Touvron H. Lavril T. Izacard G. Martinet X. Lachaux M. A. Lacroix T. . LLaMA: open and efficient foundation language models. arXiv; (2023). Available online at: http://arxiv.org/abs/2302.13971

[ref19] Tu T. Schaekermann M. Palepu A. Saab K. Freyberg J. Tanno R. (2025). Towards conversational diagnostic artificial intelligence. Nature 642, 442–450. doi: 10.1038/s41586-025-08866-7, PMID: 40205050 PMC12158756

[ref20] Wang X. Wei J. Schuurmans D. Le Q. Chi E. Narang S. . Self-consistency improves chain of thought reasoning in language models. arXiv; (2023). Available online at: http://arxiv.org/abs/2203.11171

[ref21] Wei J. Wang X. Schuurmans D. Bosma M. Ichter B. Xia F. . Chain-of-thought prompting elicits reasoning in large language models. arXiv; (2023). Available online at: http://arxiv.org/abs/2201.11903

[ref22] Yao S. Zhao J. Yu D. Du N. Shafran I. Narasimhan K. . ReAct: synergizing reasoning and acting in language models. arXiv; (2023). Available online at: http://arxiv.org/abs/2210.03629

[ref23] Zaghir J. Naguib M. Bjelogrlic M. Névéol A. Tannier X. Lovis C. (2024). Prompt engineering paradigms for medical applications: scoping review. J Med Internet Res. 26:e60501. doi: 10.2196/60501 39255030 PMC11422740

