# Supplementary material for: In the search for the perfect prompt in medical AI queries
Source: Front Artif Intell. 2025 Nov 17;8:1689178. doi: 10.3389/frai.2025.1689178 (PMC12665726; doi:10.3389/frai.2025.1689178)
Supplement: Supplementary file 1 [file Table_1.DOCX]

Supplementary Material

**Examples of prompts in different contexts**

| Prompt Type | Residual pain (6) | Actovegin in traumatic brain injury (TBI) (4) | Neurorestoration after Stroke (5) | Risks & Mitigations |
| --- | --- | --- | --- | --- |
| Zero-shot | "What are the causes of residual pain in patients with rheumatoid arthritis who are in remission?" | "What is the effect of Actovegin on the functional outcome of patients with traumatic brain injury (TBI)?" | "What are the most effective non-invasive interventions for functional recovery after an ischemic stroke?" | Risks: Vague, inaccurate, or overly general responses due to lack of context.  Mitigations: Best for simple, factual queries. For complex tasks, add context or use a more structured prompt. |
| Few-shot | "Here are two examples of patient questions about their treatment: [Example 1: 'Will this biologic medicine completely cure my arthritis?'] [Example 2: 'Why do I still have pain even though my inflammation tests are normal?']. Now, formulate an educational response for a patient asking why they should continue treatment if the disease is in remission but the pain persists." | "Here are two examples of study conclusions: [Example 1: 'Standard rehabilitation therapy leads to a mean improvement of 10.61 points on the FIMt scale.'] [Example 2: 'Adding drug X to standard therapy did not show a statistically significant improvement over placebo.']. Now, write a conclusion for the Actovegin study, comparing the FIMt score improvement in the treatment group (mean increase of 30.64) versus the control group (mean increase of 10.61)." | "Here are two examples of clinical study findings: [Example 1: 'Repetitive transcranial magnetic stimulation (rTMS) showed short-term therapeutic effects on the lower limbs.'] [Example 2: 'Physical exercise therapy improved gait speed and balance.']. Now, formulate a synthetic conclusion about the potential of combining different non-invasive therapies (e.g., rTMS with kinesiotherapy) to maximize motor recovery in chronic stroke patients." | Risks: Biased or flawed output if the provided examples are not diverse, accurate, or representative.  Mitigations: Use diverse and accurate examples. Validate the output against external sources to check for bias. |
| Ask Me Anything | "I am a rheumatologist treating patients with ankylosing spondylitis who, despite achieving biological remission on bDMARDs, continue to report pain and fatigue. What are the best diagnostic and management strategies for this residual pain, beyond anti-inflammatory treatment?" | "I am a clinical researcher planning a new trial for neuro-rehabilitative drugs. Based on the provided study on Actovegin, what are the key methodological considerations, limitations (e.g., sample size, follow-up period), and outcome measures (like FIM, GOS, mRDS) that I should account for when designing a more robust, large-scale study?" | "I am a neuro-rehabilitation therapist. Given the multitude of non-invasive interventions mentioned in the article (e.g., rTMS, tDCS, virtual reality, constraint-induced movement therapy), what are the key considerations and challenges in selecting and combining these therapies to create a personalized rehabilitation plan for a patient in the chronic phase of an ischemic stroke?" | Risks: High risk of off-topic responses, scope creep, and fabricated information ("hallucinations").  Mitigations: Implement guardrails to limit scope. Use Retrieval-Augmented Generation (RAG) to ground answers in verified data. |
| Least-to-Most | "Let's develop a management plan for a patient with rheumatoid arthritis in remission who suffers from residual pain. First, list the possible causes of this pain, differentiating between inflammatory, neuropathic, and nociplastic mechanisms, according to the article. Next, suggest assessment tools to profile the type of pain. Finally, propose a multimodal treatment strategy that includes non-pharmacological therapies and centrally-acting medications." | "Let's design a protocol for a follow-up study on Actovegin for TBI. First, define the inclusion and exclusion criteria for patients, mirroring the ones in the article (e.g., subacute TBI, GCS ≤ 12). Next, specify the treatment and control group protocols, including the dosage (200 mg Actovegin®, 2 tablets/day). Then, list all the scales used for evaluation at admission and discharge (FIM, GOS, mRDS, DRS, ADL). Finally, propose a statistical analysis plan to compare the outcomes between the two groups." | "Let's develop a research framework for evaluating a new post-stroke rehabilitation intervention. First, define the key concepts of neurorestoration, neuroregeneration, and brain repair as presented in the article's introduction. Next, list the main categories of non-invasive, non-pharmacological interventions (e.g., brain stimulation, kinesiotherapy, brain-computer interfaces). Finally, propose a systematic review methodology, similar to PRISMA, to select and evaluate relevant studies in this field." | Risks: Error propagation, where a mistake in an early reasoning step invalidates all subsequent steps.  Mitigations: Validate each step of the reasoning chain, either manually ("human-in-the-loop") or via automated consistency checks. |
| Role Assignment | "Act as a pain management specialist and provide recommendations to a rheumatologist on how to approach a patient with rheumatoid arthritis in remission who reports persistent pain (VAS score > 40 mm) and has a painDETECT questionnaire suggestive of a neuropathic component." | "Act as a rehabilitation medicine physician. A 40-year-old patient was admitted to your clinic for post-acute TBI rehabilitation, 2 months after the initial injury. Based on the findings of this study, explain the potential benefits of adding Actovegin 200 mg, 2 tablets/day, to their standard neuro-rehabilitative therapy." | "Act as a neurologist advising a rehabilitation team. Based on the evidence in this article, argue why a combined approach including physical exercise (kinesiotherapy), cognitive stimulation in an enriched environment (EE), and neuromodulation techniques like tDCS would be superior to any single therapy in promoting neuroplasticity and functional recovery in stroke patients." | Risks: Creates a false sense of authority, leading users to over-trust potentially incorrect, yet convincing, information.  Mitigations: Mandate a clear disclaimer that the AI is a simulation and its output is not a substitute for professional advice. |
| Tone | "Explain the concept of 'post-remission syndrome' to a patient in simple and empathetic language. Use an encouraging tone, avoiding complex medical terminology, to help them understand why symptoms like pain and fatigue can persist even when inflammation is controlled." | "Explain to the family of a TBI patient, in a clear and cautiously optimistic tone, the results of this study. Emphasize that while Actovegin is not a cure, the study suggests it can help improve functional outcomes like daily living activities and cognitive function when added to standard rehabilitation, but that larger studies are needed for confirmation." | "Explain to a patient who has had an ischemic stroke, using a realistic but encouraging tone, why rehabilitation is a long-term process. Emphasize, based on the article, that the window for recovery is longer than previously thought and that various non-invasive therapies, like physical exercise and mirror therapy, can help the brain reorganize and improve function, even in chronic stages." | Risks: An inappropriate tone (e.g., too casual for serious news) can trivialize information or make it inaccessible.  Mitigations: Explicitly define the target audience and purpose in the prompt to ensure the tone is appropriate for the context. |
| **Contextual Priming** | "A 45-year-old patient, diagnosed with ankylosing spondylitis 10 years ago, is in remission under treatment with TNF-alpha inhibitors (disease activity measured by ASDAS is low). However, he reports chronic widespread pain, fatigue, and sleep disturbances, which affect his quality of life. Considering that inflammatory markers are within normal limits, what diagnostic hypotheses should be considered to explain this residual pain, and what therapeutic options, other than changing the biologic therapy, would be appropriate?" | "A 33-patient study group with subacute TBI received 200 mg Actovegin®, 2 tablets/day, in addition to standard therapy, and was compared to a 41-patient control group receiving only standard therapy. The Actovegin group showed a significantly greater mean increase in the FIMt score (30.64 vs. 10.61) and FIMc score (10.94 vs. 3.41) compared to the control group (p < 0.001). Considering these results, what is the clinical significance of adding Actovegin to the treatment regimen for post-TBI patients in the subacute phase?" | "A systematic literature review identified 83 relevant papers on non-invasive, non-pharmacological interventions for recovery after ischemic stroke. The article highlights that while no single therapy can completely heal the lesions, approaches like repetitive transcranial magnetic stimulation (rTMS), electrical stimulation (ES), and enriched environment (EE) promote neuroplasticity and functional recovery. Given the complexity and diversity of these interventions, what is the article's main conclusion regarding the most promising rehabilitation strategy for the future?" | Risks: Irrelevant, contradictory, or excessive context can confuse the model, leading to inaccurate or off-target responses.  Mitigations: Provide only concise, relevant information. Structure the context clearly (e.g., with headings) to guide the model's focus. |

**
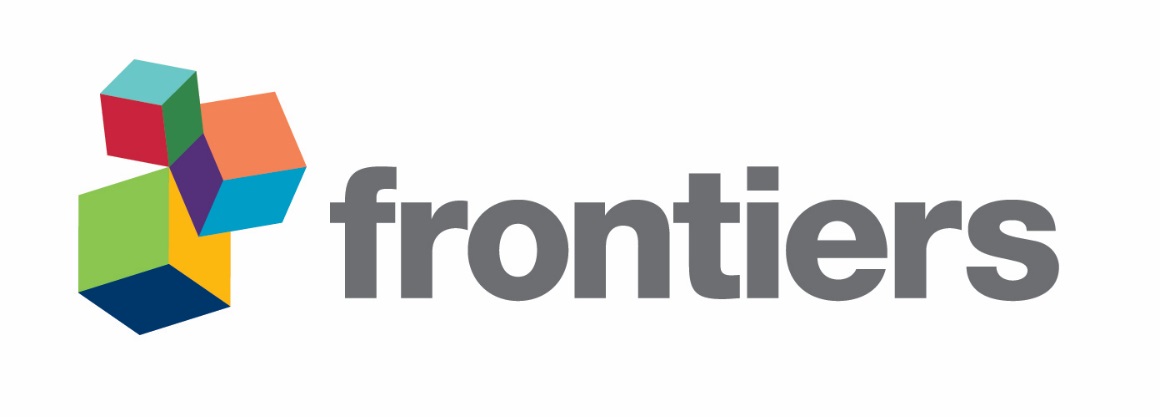
**
